# Prediction-based attention computing: a proof of concept study

**DOI:** 10.1007/s10055-025-01307-w

**Published:** 2026-01-19

**Authors:** T. Arthur, D. Borg, Y. Wang, D. Harris, S. Vine, G. Buckingham, M. Wilson, M. Brosnan

**Affiliations:** 1 Department of Public Health and Sports Sciences, School of Public Health and Sports Sciences, Faculty of Health and Life Sciences, University of Exeter, St Luke’s Campus, Exeter, Devon EX1 2LU UK; 2 Centre for Applied Autism Research, Department of Psychology, University of Bath, Bath, BA2 7AY UK

**Keywords:** Virtual reality, Predictive coding, Active inference, Adaptive training

## Abstract

Recent advancements in extended reality (XR) and data modelling present new opportunities for adaptive simulation solutions, which can measure and respond to individual neuropsychological states. However, questions remain about the optimal metrics for real-time data capture and the applicability of these solutions for enhancing user experiences. The present research examined a novel form of adaptive XR, called “prediction-based attention computing” (PbAC), which tailors simulations based on computational models of the brain and, thus, the dynamic sensorimotor processes theorised to underpin human perception and learning. Specifically, this study aimed to demonstrate whether PbAC can adaptively capture users’ internal state predictions and modulate associated neuropsychological responses. To test this, we used an XR-based racquetball paradigm, in which participants were tasked with intercepting virtual balls that emerged from different starting locations. For PbAC conditions, in-situ eye tracking data assessments were utilised to index participant’s prior beliefs and manipulate levels of expectedness (i.e., prediction error) on each trial. Various measures of predictive sensorimotor behaviour were then extracted and compared with data from probability-controlled and matched-order control conditions. Results showed that sensorimotor responses were affected by the expectedness of XR stimuli, and that clear, prediction-related biases emerged within PbAC conditions. The novel computing software also provoked marked surprisal responses on trials designed to elicit high levels of prediction error, and these surprisal effects were similar, or even greater than, those in our comparison conditions. Together, the findings provide proof of concept for PbAC and support its development within future research and technology innovations.

**Supplementary Information:**

The online version contains supplementary material available at 10.1007/s10055-025-01307-w.

## Introduction

As technological innovations like extended reality (XR) and artificial intelligence (AI) grow in prevalence and capability, there are increasing opportunities for personalised and adaptive human–computer interactions; for example, within vehicle control panels, smart phones or entertainment devices. Wearable sensors can also be used to effectively estimate psychophysiological states like anxiety (Abd-Alrazaq et al. 2023), arousal (e.g. Filippini et al. 2022; Girardi et al. 2017) and fatigue (e.g. Gholami et al. 2020; Luo et al. 2020), while data recorded from a person’s movements and interactions can be analysed to characterise task performance/learning (Harris et al. 2023; Lam et al. 2022; Rajavenkatanarayanan et al. 2018). Such data can also now be ‘fed back’ into systems that humans are interacting or coupled with, to promote adaptive changes in an environment (see *Brain Computer Interfaces*: Maiseli et al. 2023; *Adaptive XR simulations*: Zahabi et al. 2020; and *Affective Computing*: Harris et al. 2023). These capabilities have the potential to improve the efficiency and effectiveness of technological systems, and the humans operating these systems, for improved outcomes. Nonetheless, key questions remain about the applicability of these advancing digital innovations. Indeed, research must establish the optimal metrics for capturing a person’s underlying psychophysiological state, and the best methods for using these data in a meaningful way to elicit change. Similarly, focus should be placed on how new systems can be exploited to *augment* user experiences (e.g. in learning applications, therapies, and entertainment media). The present study provides an initial ‘proof of concept’ assessment for a novel digital solution, called ‘Prediction-based Attention Computing’ (PbAC), which adapts simulation features based on individualised user states and sensorimotor interactions.

One of the major challenges with designing adaptive simulations is identifying an appropriate theoretical backdrop that governs how human-system interactions are implemented. Predictive coding theories (Friston et al. 2006; Friston and Kiebel 2009), however, provide a plausible framework through which adaptive interactions could be optimised. These accounts broadly conceptualise the brain as a “prediction machine” which acts as a self-organising dynamical system that constantly seeks to minimise prediction errors (i.e., discrepancies between expected and observed sensory inputs) within its cortical networks (Clark 2013). Here, probabilistic models of the world (e.g. prior and posterior beliefs) are iteratively generated, which approximate Bayesian principles (Friston et al. 2006). These generative processes can be explained both in computational and physiological terms (e.g. as statistical models or bidirectional cascades of synaptic activity: Friston 2010; Shipp et al. 2013), and are said to underpin the fundamental attentional, cognitive, and motor functions that characterise behaviour (see ‘Active Inference’ in Parr and Friston 2019).

When coupled with more applied theories of perception and action, these predictive processing frameworks could provide clear mechanistic pathways for achieving a desired state or experience. For instance, in XR simulations, sensory stimuli could be tailored to reduce prediction errors linked with perceived lags or inaccuracies in avatar position, thereby boosting immersion and embodiment (in line with models of presence and place illusion: e.g. Witmer & Singer, 2005; Slater, 2009). Moreover, the balance of expected and unexpected stimuli could be adapted to optimise information gain, to refine attentional focus, or to reduce psychological workloads during learning or therapeutic applications (in line with computational learning models, e.g. Wolpert 2000; and skill acquisition or cognitive load theories: e.g. Wulf 2013; Mayer 1997, 2009). As such, the real-time estimation of predictive processing operations could provide unique insight into the underlying neuropsychological state of users as they interact with complex systems, and this insight could be applicable to wide-ranging contexts. Contrary to most frequently used AI approaches for modelling user states (e.g. unsupervised neural networks or data-driven classifications), such insight would be inherently aligned with contemporary models of the brain and, thus, the underlying processes theorised to underpin human sensorimotor behaviours.

Notably, research has identified various behaviours that can be approximated with ‘statistically optimal’ Bayesian computations. For example, our recent studies have focused on XR-based visuomotor tasks, which require users to intercept virtual balls with a handheld controller (Arthur et al. 2024; Arthur and Harris 2021; Harris et al. 2022; Harris et al. 2024). Here, users spontaneously exhibit gaze patterns and pupil dilation responses that are consistent with predictive coding models. Analogous prediction-related behaviours can be indexed from physiological metrics (Noordewier et al. 2021), EEG (Bekinschtein et al. 2009; Wacongne et al. 2011), and standardised cognitive paradigms (e.g. associative learning and spatial localisation tasks: Bejjanki et al. 2016; den Ouden et al. 2010; Lawson et al. 2021). However, these approaches typically rely on controlled laboratory conditions and post-hoc fitting procedures, whereby observed study data are retrospectively compared to reiterative simulated models (Hodson et al. 2023). Thus, it is pertinent to employ prospective study designs, which determine trial conditions using in-situ data assessments (e.g. appraisals of prior beliefs and prediction error). This more direct approach could not only shed light on the accuracy of these emerging theories, but could also inform the design of adaptive technologies seeking to *augment* learning and performance.

A common experimental method for assessing predictive processing is to vary probabilistic contingencies within a task. Here, the likelihood of observing expected or unexpected outcomes is manipulated in a way that makes task cues more, or less, computationally surprising (e.g. den Ouden et al. 2010; Harris et al. 2024). Such analyses assume that humans compute the causal-associative relationships between internal sensory signals and external world events, and that behavioural changes are functionally related to these ubiquitous generative models. However, predictions do not solely reflect expectations about likely sensory outcomes; rather, wider contextual variables may also be at play (Friston 2010, 2011). For example, when intercepting a ball in tennis, individuals may trade-off probabilistic elements with pragmatic factors that maximise their chance of success (e.g. by preparing for more challenging actions) in order to optimally minimise prediction error and fulfil preferred goals (Harris et al. 2024). Similarly, discernible differences in predictive processing are evident in certain user groups, such as in people experiencing anxiety (Harris et al. 2023a, b, c; Lawson et al. 2021), and in those with high autistic-like traits (Arthur et al. 2019, 2021; Karvelis et al. 2018). Therefore, adaptive human–computer interactions must account for heterogenous, task-specific neural processing that underpins natural behaviour.

**Fig. 1:**
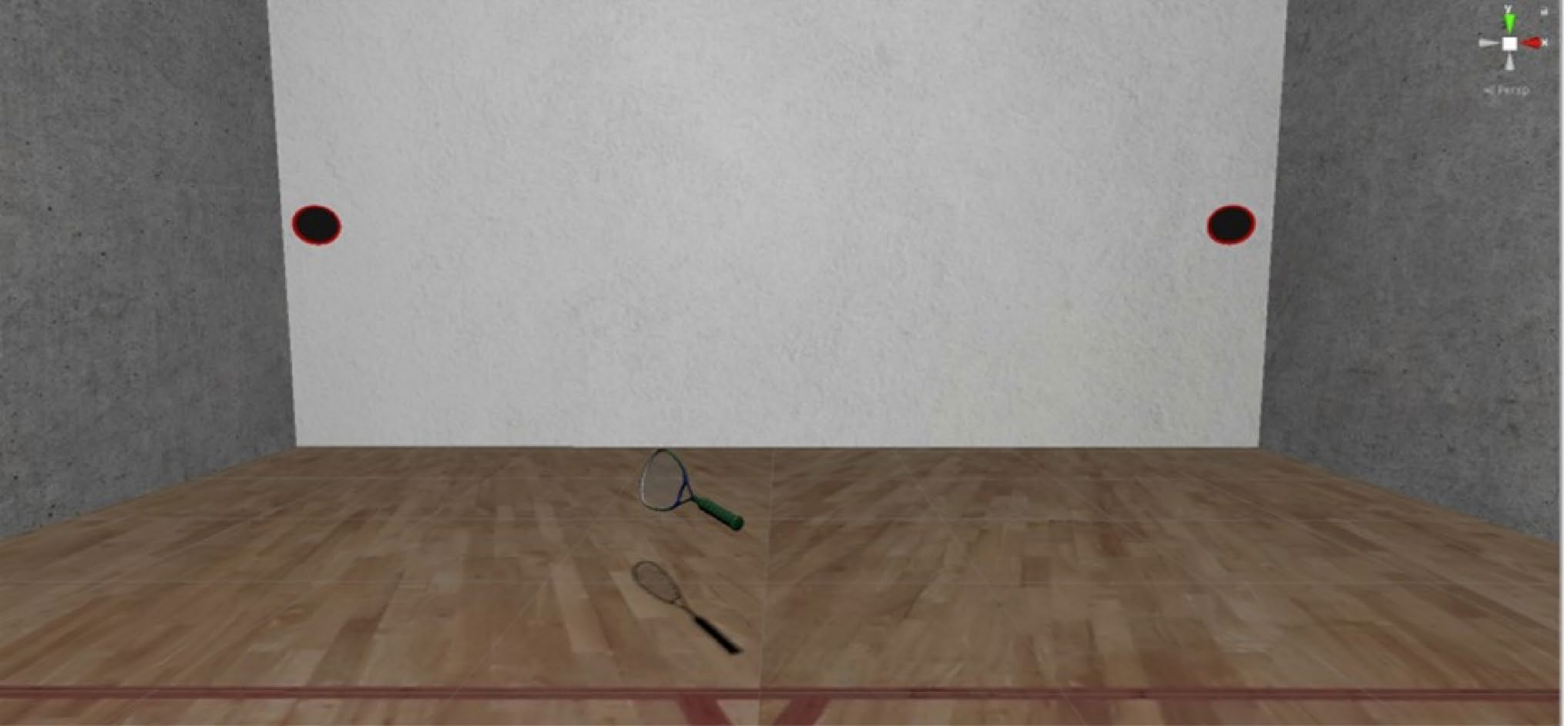
The virtual racquetball task environment. A tennis ball was projected from one of the two ‘holes’ on the front wall of the court. These holes were positioned ~ 85° of visual angle apart from each other, which encouraged participants to look towards one at a time (rather than trying to monitor both holes at once using peripheral vision). The side in which a ball appeared was either determined by prior cue-outcome associations (for probability-controlled study conditions), adaptive XR software (for PbAC study conditions), or prespecified order sequences (for order-matched study conditions). Participants stood on the centre of the red line at the start of each trial. Once a ball was projected, they attempted to hit it with the virtual racquet. For supplementary videos of this task, see: https://osf.io/37xjw/

This study examined the application of a novel PbAC solution for the monitoring and potential modulation of sensorimotor behaviours. Here, we explored whether key predictive processing operations can be accurately captured using in-situ data metrics and adaptive XR software. Specifically, anticipatory gaze responses were assessed using an established virtual racquetball paradigm (Harris et al. 2024; Harris et al. 2022), where users tried to intercept fast-moving balls that are projected from one of two possible locations (Fig. 1). Simple eye tracking analyses were performed to index dynamic state expectations (e.g. based on where they are looking or expecting the ball to be) and the hierarchical weighting of prediction errors (e.g. in relation to surprisal responses and trial-by-trial gaze changes). For the first time, these data were used to govern levels of prediction error *during the task*, such that the expectedness of ball trajectories (i.e., the degree to which sensory evidence corresponded with prior beliefs) was adapted based on estimates of users’ state predictions. Responses to expected and unexpected cues were then examined against those from probability-controlled conditions (as used in previous associative learning studies) and matched-order trial sequences. Analyses aimed to provide proof of concept for the novel PbAC solution, by demonstrating whether it can adaptively modulate users’ neuro-psychological states. If successful, we would expect participants to show divergent sensorimotor responses (e.g. poorer performance and gaze tracking) and elevated levels of surprise on trials designed to provide unexpected sensory outcomes.

In line with our research objectives, we examined the following hypotheses:

1. The impact of expectedness on sensorimotor behaviours (e.g. interception accuracy, gaze tracking responses, trial-wise learning rates) will be greater in PbAC conditions, compared to probability-controlled and matched-order trials.
2. Measures of physiological surprisal (inferred from pupil diameter responses) and prediction error (estimated from individualised computational models) will be greatest for unexpected trials in the PbAC conditions, compared to probability-controlled and matched-order trial equivalents.

## Methods

### Participants

42 participants were initially recruited for this study (14 male, 28 female; 40 right-handed, 2 left-handed; mean age: 20.74 ± 2.11 years), all of whom were above the age of 18 (range: 18–29), naïve to the study aims, and reported no prior history of cybersickness. One participant held a diagnosis of Autism Spectrum Disorder, another for Developmental Coordination Disorder, and a third disclosed to have Attention Deficit Hyperactivity Disorder. The remaining sample (*n* = 39) reported no confirmed medical or neurodevelopmental conditions that could affect their sensorimotor control. Although most participants (34/42) had used XR before, only one individual reported that they routinely use this technology, for an estimated average of 60 min per week. The remaining participants (41/42) stated that they typically do not use XR devices in their day-to-day lives. Hence, the sample were generally quite unaccustomed to the technology, and reported an average familiarity score of 2.88 out of 7 (SD = 1.19: note that a score of 1 would indicate that they are ‘*Not at all familiar*’ with XR, while a score of 7 would indicate that they are ‘*Completely familiar*’; see Sect. 2.2 below).

All participants attended a single laboratory visit and were entered into a prize draw for £100 upon study completion, if desired. Informed consent was obtained in line with British Psychological Society guidelines, and the study received approval from the University of Exeter (Public Health and Sport Sciences) and University of Bath (Department of Psychology) ethics committees. The target sample size was informed from a priori power calculations, which were conducted using data from previous related studies. Specifically, these power calculations were conducted on G*Power (Faul et al. 2007), which first converted an expected partial eta squared (*ηp*^*2*^) value into Cohen’s f and then computed the probability of detecting a true effect, given a specified sample size and significance level. Based on the previously reported effects for interceptive sensorimotor behaviours and surprisal data (in Arthur et al. 2023; Harris et al. 2022; Harris et al. 2022), a medium effect size of *ηp*^*2*^ = 0.13 was expected. From this, it was then determined that 35 participants would be sufficient to achieve at least 85% power in our primary analyses, with alpha set at *p* = 0.05. However, to account for potential data loss and outliers, we recruited an additional 7 participants. During laboratory testing, it was observed that one participant did not sufficiently follow task instructions and their data were subsequently excluded. This afforded a final sample of *n* = 41 for data analyses (see participant flow diagram in Supplementary File 1).

### Apparatus and stimuli

We broadly employed the same XR task and set-up to that of Harris et al. (2024a). This consisted of an indoor virtual racquetball environment, developed using the gaming engine Unity (v2019.3.1f; Unity Technologies, San Francisco, USA) and presented on an HTC Vive Pro Eye head-mounted display (HTC Inc., Taoyuan City, Taiwan). The Vive Pro Eye represents a high-precision, consumer-grade VR system with built-in eye tracking. Here, two infrared ‘lighthouse’ base stations act to record headset and hand controller movements at 90 Hz, while binocular pupil and gaze data were recorded at 90 Hz (with a spatial accuracy of 0.5–1.1° and latency of 10 ms). The system can accurately track kinematics in small-area movement tasks (Niehorster et al. 2017), and the SRanipal SDK enables real-time detection of gaze data (for PbAC conditions).

The virtual environment enabled participants to manually intercept balls that were projected from one of two release locations (illustrated in Fig. 1). For the current study, these two release locations were positioned 3.67 m from the centre of the front wall (~ 85° of visual angle apart), to disincentivise users from adopting a ‘visual pivot’ strategy (i.e., where participants ‘hedge their bets’ and fixate towards the centre of the front wall prior to ball release; Arthur et al. 2024). Balls were designed to resemble the size (diameter: 5.7 cm) and appearance of those in real-world tennis environments and, once released, were programmed to follow naturalistic motion dynamics (e.g. flight paths that were consistent with the effects of gravity). The side of their release was determined based on either associative cue-outcome probabilities (in block 1), PbAC software (block 2), or a prespecified trial order sequence (block 3). Participants then attempted to intercept the ball with an oval-shaped 0.6 × 0.3 m racquet, which was operated by the Vive Pro hand controller.

To enable our exploratory analyses in this study, participants were additionally presented with a demographic questionnaire and the 50-item Autistic Quotient (AQ; Baron-Cohen et al. 2001). The demographic questionnaire required participants to self-report their age, gender, and general XR usage, both in terms of their familiarity with the technology (ranked on a 7-point Likert scale from “Not at all familiar” to “Completely familiar”), and the estimated time spent gaming on XR devices per week (i.e., in their customary day-to-day lives). The AQ was then used to capture underlying differences in predictive processing that exist between individuals with varying autistic-like traits. Further details relating to this measurement tool and our exploratory analyses of individual differences can be found in Supplementary File 2.

### Procedures

Participants visited our laboratory for a single visit, during which they completed all three study conditions. Informed consent was provided upon arrival and participants were initially fitted with the VR headset and familiarised with the simulated racquetball environment. Thereafter, they received standardised task instructions (available at: https://osf.io/37xjw/), which explained the experimental protocol and their objectives. Crucially, the instructions stated that participants should aim to intercept as many balls as possible with the virtual racquet, but that it does not matter where the balls go when they are hit. Participants were also informed that the balls will usually come out of one side more than the other, but that these probabilities could change over time (in line with previous protocols: e.g. Lawson et al. 2017, 2021; Sapey-Triomphe et al. 2021). Eye tracking calibration was performed before commencement of the task, using the Vive Pro Eye’s standardised 5-point process. This process was repeated following any obvious displacements of the headset during the experiment, with no objective drift checks performed or logged. Participants were then positioned in the centre of the court, as marked with a triangle on the simulated court floor (see Fig. 1), and given a final opportunity to ask questions before beginning the racquetball game.

For the racquetball game, participants would start in the centre of the court with their hands lowered to their side. Three auditory tones would be sounded, then a ball would be projected from one of two possible release locations. There was a variable onset time delay of 0–5 s between the final auditory tone and ball release events (as in Harris et al. 2024). This delay was randomly selected from a uniform distribution and served to generate a greater implicit demand on correctly predicting the ball’s origin. A single ball would then be projected on each trial, which would travel to a point that was 1.5 m high (approximately chest height) and 0.75 m away from the court centreline (on the same side as it was initially projected from). Balls travelled at an average speed of 10 m/s and did not bounce before reaching the participant; hence, there was a high demand placed on correctly anticipating the release location. When successfully intercepted, balls would disappear and a rewarding ‘ding’ tone was played in combination with a haptic vibration from the controller. Conversely, an unrewarding ‘buzz’ was played when balls were missed, and no haptic vibrations were received.

The racquetball protocol started with ten practice trials, where 80% of balls would be projected from one ball release location and 20% would be projected from the other. While the purpose of these trials was to familiarise participants with the task, these probabilities were aligned with those in the first experimental block to establish accurate (but imprecise) prior distributions. Thereafter, they would undergo three study conditions, which were separated into distinct blocks and presented in the same order for all participants. For block 1, the *probability-controlled* trials, ball release location was determined in a manner that is akin to conventional associative learning protocols (den Ouden et al. 2010; Harris et al. 2024; Lawson et al. 2021). Specifically, 80% of balls would be projected from one side (expected trials), while 20% would be projected from the other (unexpected trials). The most likely release location was counterbalanced between participants, such that half would face more balls on their dominant side, while the other half would face more balls on their non-dominant side.

For block 2, the *PbAC* trials, ball release location was determined by participant gaze positions in the final moments before a trial. Here, real-time eye tracking data were obtained using the SRanipal SDK in Unity and fed back into the simulation using customised C# scripts (available at https://osf.io/37xjw/). As the two possible release locations were at identical heights, we specifically extracted data pertaining to the horizontal ‘in-world’ gaze coordinates (i.e. the intersection point of the gaze vector on the x-axis of the virtual environment). In effect, these data simply illustrate how far participants were gazing to the left or right of the room. No manual preprocessing of data was performed online or in real-time within Unity; rather, only raw positional coordinates generated by the Vive Pro Eye and SRanipal software were utilised within our PbAC code/software. Ball release locations were then determined by participants’ horizontal gaze position, relative to the midline of the virtual room, at 50 ms prior to trial onset (i.e. whether they were looking to the left- or right-side of the front wall just before the ball was projected). Our previous studies have shown that gaze patterns in this specific period are a valid indicator of participants’ predictions about the forthcoming trial (e.g. Harris et al. 2022; Harris et al. 2024; Harris and Arthur 2025). By choosing a timepoint that was just before the ball was projected, these indicators would reflect the dynamic nature of predictive processes (i.e. they would highlight current state expectations and would be responsive to belief updating over time). In principle, we were thus able to control whether participants subsequently observed an expected or unexpected outcome. That is, on 80% of trials, balls originated from the same side that participants were directing their gaze (expected trials), whereas balls originated from the opposite side on the remaining 20% (unexpected trials).

Finally, for block 3, the *matched-order* trials, ball release locations exactly replicated the trial-by-trial locations in block 2, irrespective of where participants were looking or the probabilistic contingencies associated with them. Consequently, expected (*n* = 40) and unexpected (*n* = 10) trials were equally distributed within blocks 1 and 2, and presented in identical pseudo-randomised order sequences (available at https://osf.io/37xjw/). However, since block 3 did not contain either probabilistic or adaptive manipulations of sensory outcomes, trials were assigned ‘expected’ or ‘unexpected’ values based on their matched order equivalents (i.e., if trial 16 in block 2 was classed as expected, then it would also be classed as expected in block 3). Hence, the only difference between each study condition was the method through which trial ‘expectedness’ was determined. Typically, each condition lasted ~ 10 min, and participants were offered a short break between blocks. During these breaks, participants were asked if they were experiencing any adverse effects in VR (e.g. cybersickness) – the racquetball task would be terminated in such cases. However, no adverse effects were reported by participants during breaks in this study. Once concluded, participants completed the demographics questionnaire and AQ. Together, this culminated in a lab visit of ~ 60 min per person.

### Data analysis

#### Sensorimotor measures

Interception rate (%) was derived from the proportion of trials in which participants successfully made contact between the virtual ball and racquet. Predictive gaze behaviours were then examined from eye tracking data. During these offline analyses, data were processed using customised MATLAB scripts (see https://osf.io/37xjw/), which initially denoised recording signals using a three-frame moving median filter and a second-order lowpass Butterworth filter (cutoff: 15 Hz; Fooken and Spering 2020). Since data recordings were obtained at a constant frame rate of 90 Hz within Unity, we did not resample any of the eye tracking data analysed in this study. Moreover, it must be noted that this filtering and preprocessing of data did not occur in the real-time PbAC conditions (e.g. when determining ball release locations within Unity). Rather, for our subsequent analyses of study data, predictive gaze locations were taken from the filtered, horizontal ‘in-world’ coordinates at the onset of trials (i.e., the intersection point of the gaze vector with the virtual environment, averaged over the final 50 ms before ball release; Harris et al. 2024), with coordinates > 0 classed as a ‘right predictions’ and coordinates < 0 classed as a ‘left predictions’ (note that a value of 0 would represent a point that is precisely in the middle of the two release locations). For the purposes of manipulation checks (outlined in Sect. 2.4.4), coordinates from the probability-controlled conditions were converted based on the veridical distribution of ball release locations. Here, more positive values were assigned to gaze positions that were towards the most probabilistically likely side of the racquetball court.

The accuracy of in-flight tracking behaviours was assessed from our gaze orientation data. For these calculations, single unit vectors corresponding to cyclopean direction were extracted for gaze and ball, thereby providing yaw and pitch rotation values that were relative to the origin of the VR headset. The degree to which participants successfully tracked the ball could then be examined by quantifying the Euclidean distance, or angular error, between the gaze and ball vectors (as in Arthur et al. 2021). Accordingly, we computed the minimum gaze-ball error between trial onset and the point of ball contact for each trial. When participants failed to intercept the ball, error values were analysed up until the point where the ball first passed the racquet. For this metric, lower values would indicate closer in-flight tracking of the ball. Alternatively, poorer gaze tracking profiles have been shown to correspond with unexpected ball flight trajectories (Arthur et al. 2021, 2024; Arthur and Harris 2021).

#### Pupillometry measures

To examine surprisal responses, we analysed changes in pupil diameter during unexpected trials. Binocular data were extracted from the VR eye tracker to provide estimates of pupil diameter, in millimetres at 90 Hz. Data for all trials were initially checked for missing data and blinks (which were padded by 150 ms and replaced by linear least squares interpolation; Relaño-Iborra and Bækgaard 2020), before being filtered using a 10 Hz low-pass Butterworth filter. Diameter values were processed for both eyes and then averaged into a mean. Thereafter, we identified the peak value that occurred following the release of the ball on each trial (up until 3 s after the ball had reached the player), to capture noradrenaline-related changes in phasic activity that occur in response to computationally salient stimuli (Harris et al. 2022; Joshi and Gold 2020; Nassar et al. 2010). The luminance of the XR environment was held constant during the study, and visual stimuli was mirrored for the left and right sides of the racquetball court (such that retinal illumination would not be disproportionately altered by gaze shifts to either side of the workspace). To account for changes in arousal, we subtracted ‘baseline’ pupil values during the 200 ms before ball release from the peak diameter scores. A baseline period of 200 ms was used to replicate previous work (Lawson et al. 2021; Harris et al. 2022) and to capture the anticipatory fluctuations in arousal that occur in the final moments before trial onset (e.g. due to changing expectations that ball release is imminent or that this event has already occurred and the participant has missed the cue). As a result, higher pupil diameter values were interpreted to reflect greater levels of surprise in this context.

#### Computational modelling and parameters

Although the predictive beliefs and errors thought to drive perception and action in this task are not directly observable, they can be inferred using computational modelling. To this end, we calculated model-based estimates of learning rate and prediction error for each participant and condition, using well-established statistical methods that have previously been applied in this task (Harris et al. 2022; Harris et al. 2024).

First, it was necessary to identify the most appropriate model that could be fit to our data. We used Bayesian model selection to compare the relative probabilities of two families of learning model: the Hierarchical Gaussian Filter (HGF; Mathys et al. 2014) and traditional associative learning alternatives (the Rescorla-Wagner model: Rescorla and Wagner 1972; and Sutton K1 model: Sutton 1992). While these methods both conceptualise predictive beliefs as dynamic, generative representations that are sensitive to prediction errors and learning rate scalars, the means through which these beliefs are updated are notionally different. Specifically, belief updating in associative learning models is assumed to be proportional to preceding prediction errors and a stable learning rate, whereas the HGF assumes learning rates to be highly changeable and influenced by hierarchical beliefs and uncertainties (Mathys et al. 2014). These dynamic HGF parameters are modelled as Gaussian random walk variables, such that the trial-wise evolution of predictions in this study will occur via Gaussian probability distributions over beliefs about a ball’s upcoming trajectory. We examined three variants of HGF models: one where learning rate scalars solely reflect participants’ evolving uncertainty about a ball’s likely release location (two-level HGF); another where scalars are also sensitive to how volatile or changeable the environment is inferred to be (three-level HGF); and a final one that accounts for varying beliefs about the rate of change in their environment over time (four-level HGF).

To identify the most appropriate model, the five potential options described above were fit to participant gaze data using an ‘observing the observer’ approach (Daunizeau et al. 2010). Here, trial-by-trial sensory observations (i.e., whether a ball followed a left- or right-sided trajectory) and action responses (i.e., whether gaze was directed to the left or right side of the court) were used to estimate a perceptual model for each computational method. To this end, we effectively used known study data to approximate the underlying inference processes of participants. Starting parameters for each model are specified in Supplementary File 3 and were determined in accordance with Harris et al (2023a, b, c). Specifically, free value beliefs about likely ball release location and learning rate were set at neutral starting values in the associative learning models. Conversely, prior means in the HGF models were chosen to represent values under which an ideal Bayesian agent would experience the least surprise about its sensory inputs. These values are listed in Supplementary Table 2 and were determined by running simulations through real trial sequences in the experiment (and using the resultant posterior means from these ideal observer models). In light of the relatively few trials in our study, Kappa values were fixed at 1 with a variance of 0, to reduce model complexity. However, to allow for individual differences, starting beliefs were given a wide variance in all five models and were relatively uninformative. Model fitting and comparison procedures were performed using the TAPAS (Frässle et al. 2021) and HGF (Mathys et al. 2011, 2014) toolbox.

Once fitted to the data, the five competing models were compared through Bayesian model selection (Rigoux et al. 2014) using the SPM12 toolbox (https://www.fil.ion.ucl.ac.uk/spm/software/spm12/). This process would provide a probability-based estimate of whether a given model outperforms the other comparisons, termed the protected exceedance probability. It also enabled quantifications of relevant trade-offs in terms of relative accuracy and complexity: namely, log model evidence (LME) scores for each compared option. To foreshadow the results, the three-level HGF model was found to provide the most plausible explanation for participants’ predictive gaze behaviours in this study (as in our previous related research: Harris et al. 2022; Harris and Arthur 2025). Accordingly, we fit participants’ predictive gaze (left or right side) and ball release location (left or right side) data from across all experimental trials using this model. Here, gaze and ball release data were assigned a binary value based on their counterbalanced condition, such that a value of 1 was assigned for gaze and ball locations towards the most probable side (defined from block 1) and a value of 0 was assigned for those positioned on the least likely side. Since both the sensory inputs and responses are known for individuals in this task, the model could then estimate various participant-specific parameters and dynamic belief trajectories (relating to an individual’s inferences about the world, their uncertainty, and the degree to which they perceive environmental states to be changing). Such computations are based on generative assumptions, and the notion that predictive processes approximate statistically optimal principles (in a manner that is consistent with Bayesian inference theories). Further details about the mechanics of the model are described in Mathys et al. (2014).

To address our first research hypothesis, we extracted the learning rate parameter *α*_*2*_ from the fitted HGF model. This parameter captures the amount of weight ascribed to a recent sensory observation in relation to inferred cue-outcome relationships. In the current context, *α*_*2*_ values provide an indication as to how quickly participants are updating their beliefs about a ball’s likely release location on each trial. For interpretation purposes, negative learning rates (i.e., belief trajectories that transition towards a binary value of 0) were transformed into positive values. Consequently, higher *α*_*2*_ scores would reflect greater updating of prior beliefs in response to expected or unexpected trial outcomes. To address our second research hypothesis, we extracted precision-weighted prediction error parameters ε_2_ and ε_3_ from unexpected trials. These parameters reflect the degree of surprise relating to dynamic cue-outcome beliefs (ε_2_) and expectations about environmental change (ε_3_). Indeed, for the current study paradigm, higher model-based estimates of prediction error have been shown to correspond with unexpected ball trajectories (Arthur and Harris 2021) and elevated physiological surprisal responses (Harris et al. 2024).

#### Statistical analysis

Outcome variables were screened for missing values and outliers, with any trials containing > 25% of missing data discarded and any extreme scores > 3 SDs away from the mean replaced with a Winsorized substitute (i.e. a value that is 1% larger or smaller than the next most extreme score). Participants whose data contained more than a third of removed trials (e.g. due to missing or invalid eye tracking values) were excluded from the respective analysis. Sensitivity checks established that results were unchanged without Winsorization.

Cleaned data were then entered into a series of manipulation checks. Firstly, to establish if participants were exhibiting anticipatory gaze behaviours that are consistent with an internal generative model, we conducted a one-sample t-test of median predictive gaze locations from the probability-controlled conditions in block 1. Thereafter, a dependent t-test compared interception rates between expected and unexpected trials, to establish if there were prediction-related biases evident in participants’ visuomotor task performance.

To address our first hypothesis a 3 × 2 ANOVA examined the effects of condition (probability-controlled, PbAC, matched-order control) and expectedness (expected, unexpected) on interception rate, gaze tracking error, and learning rate outcomes. To address our second hypothesis, pupillometry and model-based prediction error data from unexpected trials were compared between conditions, using a one-way ANOVA. Significant main effects were followed-up with post-hoc t-tests, which were adjusted using the Bonferroni correction. For significant interaction effects, our post-hoc t-tests focused on difference scores (Δ) between expected and unexpected trials within-subjects (as in Arthur et al. 2021). Finally, Pearson’s Correlation analysis explored relationships between interception rate and measures of gaze tracking error, pupil diameter, and modelled estimates of prediction error on unexpected trials. For non-parametric data, correlations were examined using Spearman’s Rho analysis.

The strength of evidence for all statistical tests were interpreted using Bayes Factors (BF_10_), with moderate support for the alternative hypotheses set at BF_10_ > 3 and strong support indicated by BF_10_ > 10. Inferences relating to our main hypotheses were predominantly guided by frequentist test statistics, based on scrutiny of p-values and associated effect sizes (e.g. partial eta squared for ANOVAs, Cohen’s *d* and 95% confidence intervals for this statistic in t-tests). However, additional analysis of Bayes Factors enabled probabilistic interpretations to be made, while allowing for the direct quantification of evidence in favour of both alternative *and* null models. All analyses were performed using JASP (v0.12.2), with statistical significance accepted at *p* < 0.05. Cleaned study data are available at: https://osf.io/37xjw/.

## Results

### Descriptive statistics and manipulation checks

**Fig. 2:**
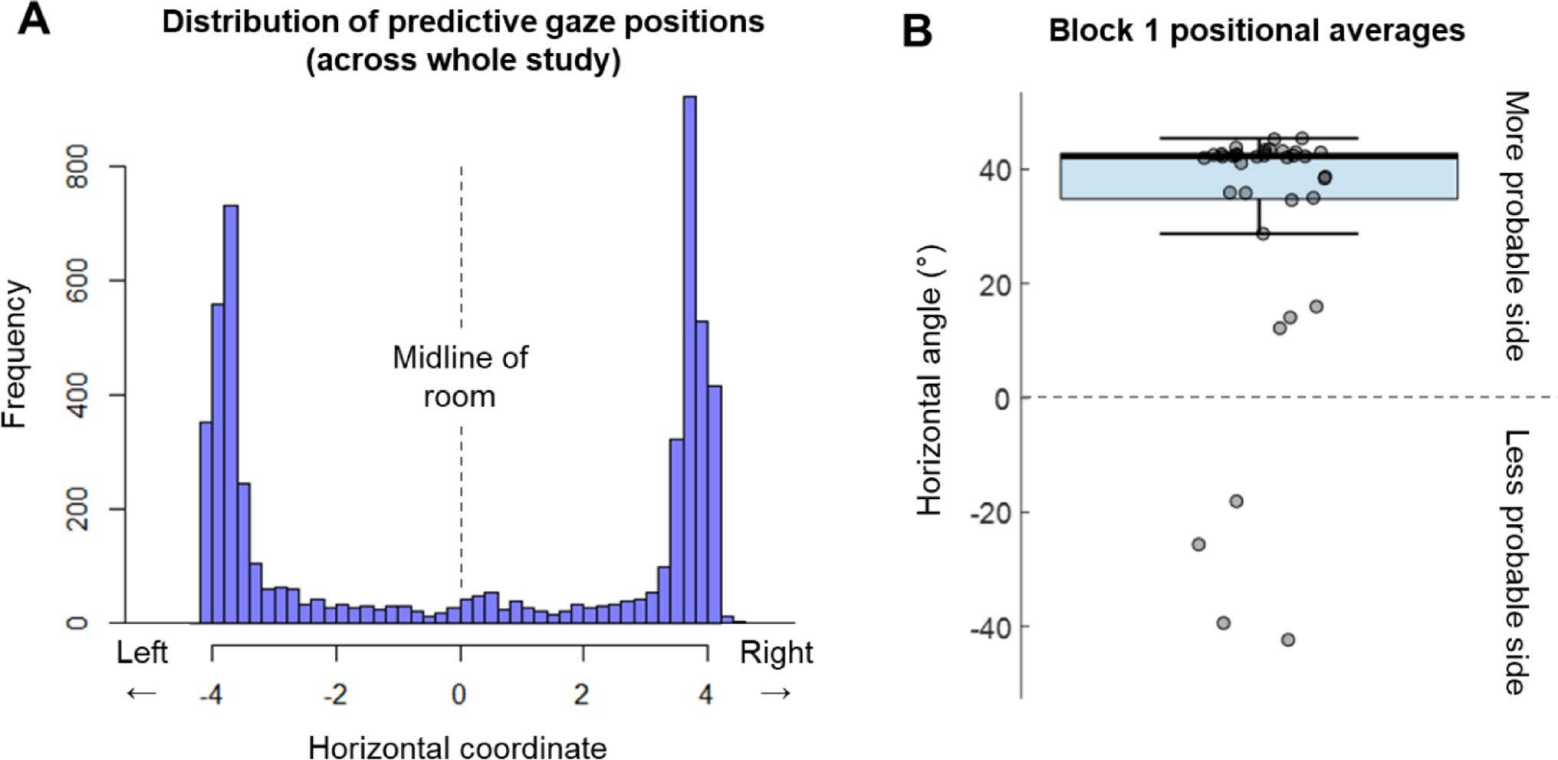
Anticipatory gaze behaviours. Panel **A** shows the horizontal ‘in-world’ gaze coordinates recorded at 50 ms before ball release on each trial, with higher values corresponding to more rightward locations. Panel **B** shows averages from this same timepoint, but for trials in the probability-controlled conditions only. Here, positional data have been converted into angular coordinates, with greater values assigned to gaze locations that were more towards the most probabilistically likely side of the court. Dots represent medians for each participant. The box plot illustrates the median and the upper and lower quartiles of the overall sample. In both panels, a value of zero would represent a point that is precisely in the middle of the two ball release locations

Five participants were excluded due to excessive loss of eye tracking data (see Supplementary File 1). Remaining data were deemed of sufficient quality for analyses. Descriptive statistics showed that participants directed their gaze 41.17° from the centre of the racquetball court, on average, in anticipation of ball release. Gaze was located within 0.4 m (5.71°) of the midline on 2.59% of trials, indicating that predictive strategies were being adopted and that visual pivot strategies were rare (see Arthur et al. 2024). Figure 2A highlights a bimodal distribution of data, with participants generally directing their gaze to one of the two prescribed ball release locations on each trial (as opposed to the centre of the front wall). Average predictive gaze locations were significantly greater than zero during block 1 (*t*(35) = 7.69, *p* < 0.001), with a large effect size evident (*d* = 1.28, 95% CI [0.83, 1.72]). Bayes Factor analysis provided strong evidence in favour of this effect (BF_10_ = 2.48 × 10^6^), and Fig. 2B illustrates that 32/36 participants fixated towards the high-probability side of the court. Moreover, across the whole experiment, participants intercepted a significantly higher proportion of balls that were from the expected, as opposed to unexpected, side (*t*(35) = 9.46, *p* < 0.001). Again, a large effect size was evident (*d* = 1.58, 95% CI [1.08, 2.06]) and Bayes Factor analysis provided strong supporting evidence (BF_10_ = 2.70 × 10^8^). Thus, clear anticipatory biases were evident in sensorimotor behaviour, and the planned analyses of trial expectedness and surprisal responses were considered appropriate.

### Computational model comparisons

**Fig. 3:**
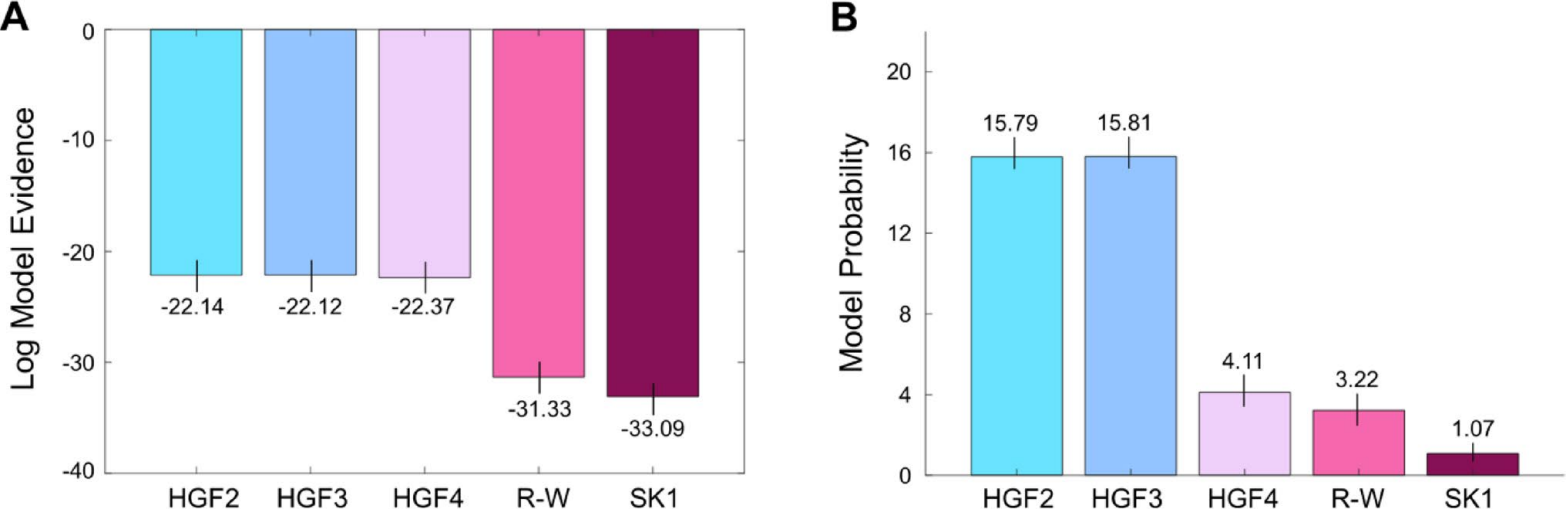
Model fitting and comparison results. Panel **A** illustrates Log Model Evidence values for each computational model. Here, the three-level Hierarchical Gaussian Filter (HGF3) marginally outperforms its two- (HGF2) and four-level (HGF4) alternatives and clearly outperforms the Rescorla-Wagner (R-W) and Sutton K1 (SK1) models. This was supported by protected exceedance probability scores, which were highest for the HGF3 (0.50), near equivalent for the HGF2 (0.49), and much lower for the HFG4, R-W, and SK1 models (all 0.003). Panel** B** then plots the probabilities of each model in the sample population, based on Bayesian model selection. Again, the highest values are shown for the HGF3 model, which was subsequently used for the extraction of learning rate and prediction error parameters

To determine the most appropriate computational model that could be fit to our data, we used Bayesian model selection to compare the two associative learning models (Rescorla-Wagner and Sutton K1) and three HGF models (Mathys et al. 2011, 2014) described in our methods. Results indicated that the three-level HGF was the most plausible model for our study data, based on the fact that it produced the highest log-model evidence (Fig. 3) and protected exceedance probability value (0.50). Notably, this model was 9963.66 times more likely than the R-W, and 57895.79 times more likely than the SK1 models. As a result, it can be assumed that Bayesian inference captured individual learning profiles most successfully in our task, and that participants were using dynamic beliefs about probabilistic uncertainty to modify behaviour (as in Arthur and Harris 2021; Harris et al. 2022; Harris and Arthur 2025).

It must be acknowledged that the three-level HGF only marginally outperformed the other HGF methods (Fig. 3), with Bayes Factor calculations indicating that it was only slightly more likely than its two-level (BF_10_ = 1.01) and four-level (BF_10_ = 1.28) equivalents. Nonetheless, previous studies have also demonstrated that the 3-level model is more plausible than a four-level HGF in this task (Harris et al. 2022; Harris and Arthur 2025), and that pupil-based surprisal responses align with *α* and ε parameters extracted from this given method (Harris et al. 2022). Moreover, research has linked changes in predictive gaze (Arthur and Harris 2021; Harris and Arthur 2025) and pupil diameter (Lawson et al. 2021) responses to the dynamic encoding of volatility beliefs, which are represented at the third level of HGF models. Consequently, for comparisons of learning rate and prediction error responses between conditions, we opted to extract parameters from the three-level HGF, as described in our methods.

### Impact of trial ‘expectedness’ on predictive sensorimotor behaviours

As detailed in our methods, data were separated for expected and unexpected trials across each study condition. Here, ‘expectedness’ was determined by veridical cue-outcome associations for the probability-controlled conditions and by adaptive eye-tracking software for the PbAC conditions. For the order-matched conditions, however, trial expectedness did not necessarily align with participants’ internal beliefs or their tracking of cue-outcome associations. Rather, ball release locations replicated those in the previous PbAC conditions and expectedness labels for each trial were simply inherited from this preceding block (in order to isolate possible sequencing effects).

**Fig. 4:**
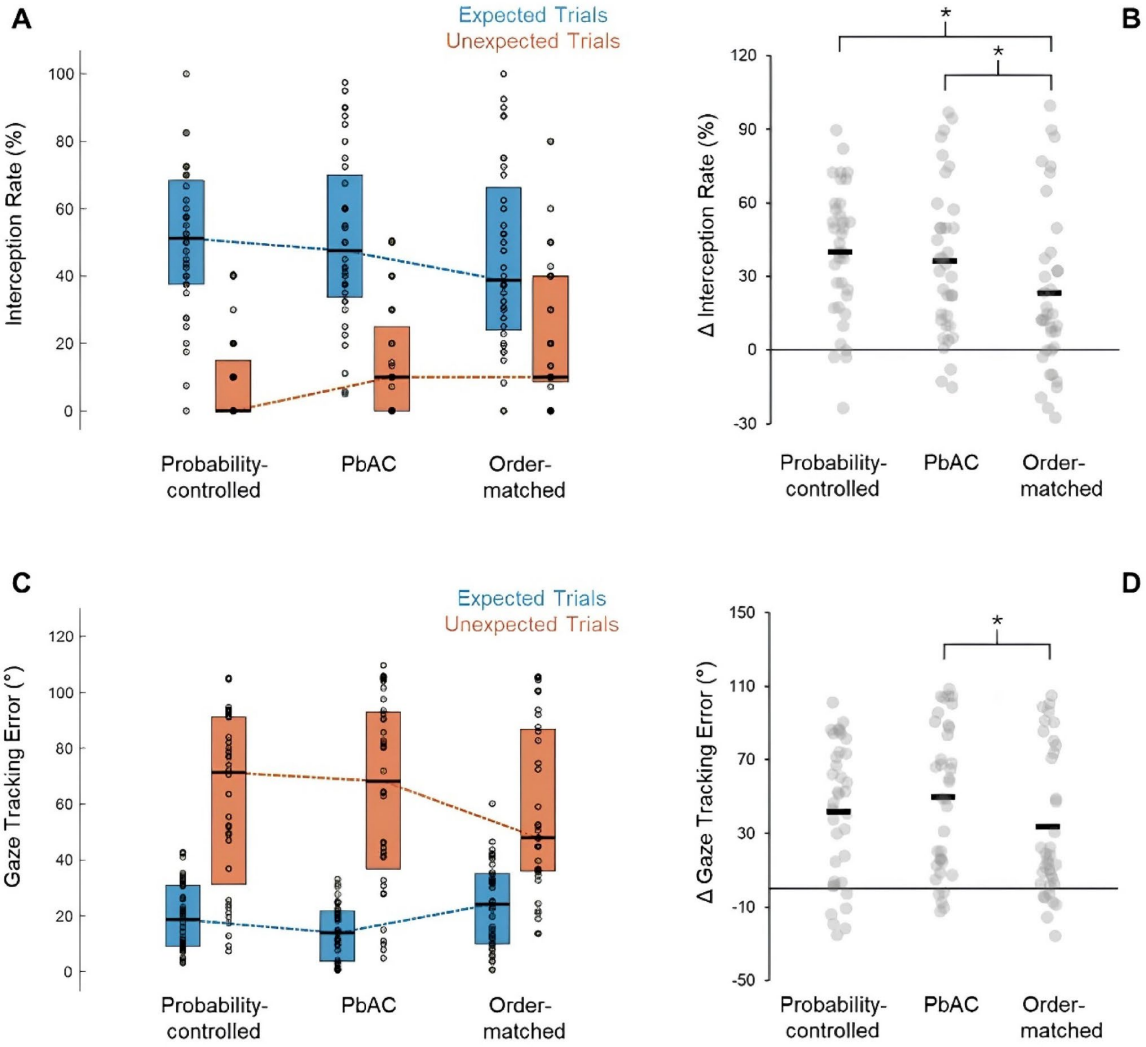
Patterns of predictive sensorimotor behaviour during each of the three study conditions. Circles represent mean values for each individual participant. Bars in panel **A** and **C** represent the median averages (black lines) and the upper and lower quartiles for each condition. Black lines in panels **B** and **D** conversely represent the mean averages for each overall condition, with higher Δ values illustrating larger differences between expected and unexpected trials (i.e., greater behavioural surprise). Asterixis denote significant between-condition differences (*p* < .05). PbAC: Prediction-based Attention Computing

For interception rate, ANOVA revealed null effects of condition (*F*(2,70) = 1.34, *p* = 0.27, *ηp*^*2*^ = 0.04, BF_10_ = 0.17) but a significant condition-by-expectedness interaction (*F*(2,70) = 9.00, *p* < 0.001, *ηp*^*2*^ = 0.20, BF_10_ = 265.87). There was a large effect for trial expectedness, as described above, however differences between expected and unexpected trials (Δ interception rate) were reduced in matched-order trials, compared to probability-controlled (*t*(35) = 3.34, *p* < 0.01) and PbAC (*t*(35) = 3.38, *p* < 0.01) conditions. Moderate effect sizes emerged for both comparisons (vs probability-controlled: *d* = 0.56, 95% CI [0.20, 0.91]; vs PbAC: *d* = 0.56, 95% CI [0.21, 0.91]), and Bayes Factor analysis provided strong evidence in support of these effects (BF_10_ = 17.21 and BF_10_ = 18.70 respectively). There were no significant differences between probability-controlled and PbAC conditions (*t*(35) = 0.76, *p* = 0.45), with expectedness having a similar effect on task performance (*d* = 0.13, 95% CI [-0.20, 0.45]; Fig. 4B). Here, Bayes Factor analysis provided strong evidence in favour of a null effect (BF_10_ = 0.24).

For gaze tracking error, there were significant effects for trial expectedness (*F*(1,35) = 52.46, *p* < 0.001, *ηp*^*2*^ = 0.60, BF_10_ = 570,261.76), null effects for condition (*F*(2,70) = 0.44, *p* = 0.65, *ηp*^*2*^ = 0.01, BF_10_ = 0.07), and a significant condition-by-expectedness interaction (*F*(2,70) = 4.64, *p* = 0.01, *ηp*^*2*^ = 0.12, BF_10_ = 20.81). Tracking error was lower in expected than in unexpected trials (Fig. 4C). However, the magnitude of difference between expected and unexpected trials (Δ tracking error) was significantly smaller in the matched-order control trials compared to PbAC conditions (*t*(35) = 3.96, *p* < 0.001), with a moderate effect found (*d* = 0.66, 95% CI [0.30, 1.02]). Bayes Factor analysis provided strong further support for this effect (BF_10_ = 79.86). Notably, Δ tracking error values did not significantly differ between probability-controlled and either PbAC (*t*(35) = 1.41, *p* = 0.17) or matched-order (*t*(35) = 1.37, *p* = 0.18) trials. Effect sizes of *d* = 0.24 (95% CI [-0.10, 0.57]) and *d* = 0.23 (95% CI [-0.10, 0.56]) were found respectively, and Bayes Factor analysis provided support for a null effect in both cases (BF_10_ = 0.44 for probability-controlled vs PbAC conditions; and BF_10_ = 0.42 for probability-controlled vs matched-order conditions).

Lastly, the *α2* learning rate parameter was extracted from the three-level HGF model for each individual participant, with group means proving relatively imprecise in relation to both expected (probability-controlled: *M* = 0.05, 95% CI [0.01, 0.09]; PbAC: *M* = 0.00, 95% CI [-0.01, 0.003]; matched-order: *M* = 0.01, 95% CI [0.00, 0.03]) and unexpected trials (probability-controlled: *M* = 0.17, 95% CI [0.01, 0.34]; PbAC: *M* = 0.05, 95% CI [0.02, 0.07]; matched-order: *M* = 0.06, 95% CI [0.00, 0.11]). ANOVA revealed a significant effect of trial expectedness for this parameter (*F*(1,35) = 9.72, *p* < 0.01, *ηp*^*2*^ = 0.22, BF_10_ = 4.76). The rate at which participants updated their prior beliefs about likely ball location (and thus, the rate at which they updated their predictive gaze behaviours) was higher for unexpected, as opposed to expected, trials (see Supplementary Fig. 3). However, null effects for condition emerged (*F*(2,70) = 2.45, *p* = 0.09, *ηp*^*2*^ = 0.06, BF_10_ = 0.74) and there were no significant interactions present (*F*(2,70) = 1.47, *p* = 0.24, *ηp*^*2*^ = 0.04, BF_10_ = 0.34).

### Surprisal responses for unexpected trials

**Fig. 5:**
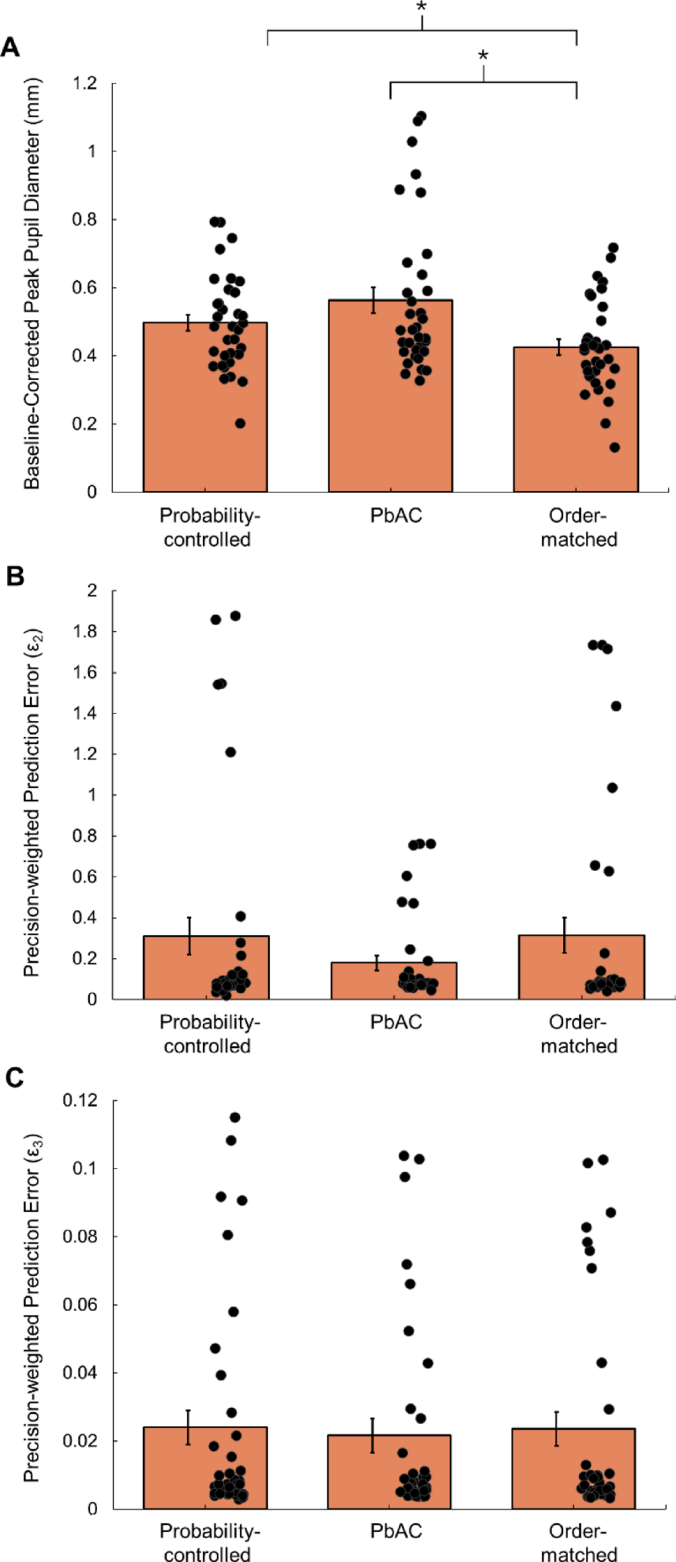
Surprisal responses on unexpected trials during each of the three study conditions. Asterisks denote significant between-condition differences (*p* < .05). Circles represent mean values for each individual participant, bars represent means (± standard error) for each condition. In all three panels, higher data values are indicative of greater ‘surprisal’. PbAC: Prediction-based Attention Computing

Two further participants were excluded from pupillometry analysis due to loss of baseline data (i.e., during the 200 ms before ball release; see Supplementary File 1). When examining cleaned data from unexpected trials only, ANOVA revealed a significant effect of study condition on participants’ peak pupil dilation responses (*F*(2,66) = 11.48, *p* < 0.001, *ηp*^*2*^ = 0.26, BF_10_ = 431.69). Specifically, peak pupil diameter was significantly higher in probability-controlled conditions than in the matched-order control block (*t*(33) = 2.99, *p* = 0.01; Fig. 5). Here, a moderate effect was found (*d* = 0.51, 95% CI [0.15, 0.87]), which was supported by Bayes Factor analysis (BF_10_ = 7.52). Similarly, peak pupil diameter values were significantly higher in PbAC conditions than in the matched-order block (*t*(33) = 4.51, *p* < 0.001; Fig. 5), as evidenced by a large statistical effect (*d* = 0.77, 95% CI [0.38, 1.15]) and strong further support from Bayes Factor analysis (BF_10_ = 314.44). When accounting for multiple comparisons, no significant differences between probability-controlled and PbAC trials emerged (*t*(33) = 2.12, *p* = 0.04) and a small effect of *d* = 0.36 (95% CI [0.01, 0.71]) was recorded. Although peak pupil diameter was slightly higher, on average, in the unexpected PbAC trials (Fig. 5A), Bayes Factor analysis provided only weak, anecdotal evidence in support of an effect (BF_10_ = 1.33).

Model estimations for precision-weighted prediction error were also extracted for unexpected trials across the probability-controlled (ε_2_: *M* = 0.31, 95% CI [0.13, 0.49]; ε_3_: *M* = 0.02, 95% CI [0.01, 0.04]), PbAC (ε_2_: *M* = 0.18, 95% CI [0.11, 0.25]; ε_3_: *M* = 0.02, 95% CI [0.01, 0.03]), and matched-order (ε_2_: *M* = 0.32, 95% CI [0.14, 0.49]; ε_3_: *M* = 0.02, 95% CI [0.01, 0.04]) conditions. ANOVA indicated that these parameters did not significantly differ between study conditions, both in relation to level two (*F*(2,70) = 1.00, *p* = 0.37, *ηp*^*2*^ = 0.03, BF_10_ = 0.22) and level three (*F*(2,70) = 0.06, *p* = 0.94, *ηp*^*2*^ = 0.002, BF_10_ = 0.09) of the HGF model. However, given that there was generally very little variability evident in our dataset (see Figs. 5B and C), it is likely that the parameters may have lacked sensitivity in the current study.

### Individual differences and correlations

Figures 4 and 5 revealed significant inter-individual heterogeneity for sensorimotor outcomes. To explore these individual data patterns, correlations were first examined between task performance and predictive sensorimotor responses during unexpected trials. Results showed that gaze tracking error was significantly related to interception rate in all three study conditions (block 1: *R*_*s*_ = − 0.34, *p* = 0.04, BF_10_ = 2.09; block 2: *R*_*s*_ = − 0.67, *p* < 0.001, BF_10_ = 1619.51; block 3: *R*_*s*_ = − 0.45, *p* < 0.01, BF_10_ = 9.51), with correlations proving strongest in PbAC trials. A weak correlation emerged between peak pupil diameter and interception rate in the PbAC block (*R*_*s*_ = 0.37, *p* = 0.03), though data provided only anecdotal support for this effect (BF_10_ = 2.90) and must be interpreted with caution. Null relationships arose between pupil diameter and interception rate for the probability-controlled (*R*_*s*_ = -0.07, *p* = 0.70, BF_10_ = 0.25) and matched-order (*R*_*s*_ = 0.20, *p* = 0.27, BF_10_ = 0.40) trials. Hence, while correlations with physiological surprise were lacking, it was evident that the participants who tracked virtual balls more closely on unexpected trials tended to also perform more successfully in these instances.

## Discussion

This study evaluated the application of a novel, prediction-based attention computing (PbAC) solution for adaptive XR simulations. Here, we used a naturalistic visuomotor task to examine the solution’s capabilities for monitoring and influencing predictive processing, relative to probability-controlled and matched-order XR conditions. By comparing against probability-controlled conditions, we could test whether established expectation- and surprise-related responses are captured similarly between PbAC and conventional research methods (where standard associative learning paradigms are typically employed). By examining PbAC against simple order-matched trials, we could then infer whether observed effects specifically relate to the encoding of generative state beliefs and expectations, as opposed to heuristic strategies or sequence-specific adaptations in motor behaviour. Results showed that interception abilities and gaze tracking responses were affected by the expectedness of VR stimuli, and that prediction-related biases emerged within the PbAC conditions. The novel PbAC solution also elicited marked surprisal responses on trials containing adaptive unexpected stimuli, and levels of surprise were similar or even greater than those in our comparison conditions. Together, these findings provide proof of concept for PbAC and support its development within future research and technology innovations.

Results provided mixed support for our first hypothesis: that trial expectedness would have a greater impact on predictive sensorimotor behaviours within the PbAC environment, compared to the study’s comparative conditions. On one hand, greater expectation-related effects were observed for both interception rate and gaze tracking data in PbAC trials than for order-matched equivalents (where trial ‘expectedness’ was not defined or adapted based on participants’ expectations or beliefs). However, these prediction-related differences were not significantly larger than those recorded in the probability-controlled conditions, where trial expectedness was determined by preprogrammed cue-outcome contingencies (as in conventional associative learning paradigms: den Ouden et al. 2010; Harris et al. 2024; Lawson et al. 2021). Notably, anticipatory patterns of sensorimotor behaviour were evident in all three study blocks, with participants predictively shifting their gaze and intercepting a higher proportion of expected (as opposed to unexpected) balls. Relationships between task performance and gaze tracking were reliably detected, and trial-wise learning rates were higher following unexpected sensory observations (in line with Bayesian learning models, e.g. Courville et al. 2006; Körding et al. 2007). The fact that sensorimotor behaviours were sensitive to probabilistic components of the task in this way lends support to models of active inference and generalised free energy (e.g. Parr and Friston 2019). Null differences between PbAC and probability-controlled conditions then imply that the novel adaptive XR solution captured these underlying generative processes, in a similar way to more established associative learning paradigms.

Results also provided partial support for the study’s second hypothesis: that surprisal and prediction error responses will be greatest for unexpected trials in the PbAC block. Again, our adaptive XR approach proved superior to the non-adaptive control conditions. Indeed, when focusing on trials that were intended to elicit high levels of prediction error, surprisal-based changes in pupil diameter were significantly more pronounced in the PbAC block than in the matched-order equivalent (block 3, where unexpected trial labels were simply inherited from the preceding study block). Such differences were not observed for model estimates of prediction error, though there were null expectancy-related effects for these parameters across the study. Notably, pupil dilation data were similar between PbAC and probability-controlled conditions, indicating that a-priori manipulations of cue-outcome probabilities were able to elicit comparable surprisal responses to our adaptive software. These null effects likely reflect task-specific features, which imposed that the optimal strategy for minimising prediction error could largely relate to probabilistic beliefs (e.g. about a ball’s most likely release location), as opposed to more general and pragmatic motor strategies (e.g. heuristic actions that maximise chances of success, *irrespective of task probabilities*). Future research could therefore explore a wider range of sensorimotor conditions, where active inference is determined by multifaceted internal beliefs and contextual variables (e.g. the expected costs, risks, or challenge associated with actions; Friston et al. 2016; Harris et al. 2024).

Nonetheless, the fact that PbAC provoked similar user responses to probability-controlled conditions is practically significant. Indeed, although probability-controlled parameters can be feasibly programmed for controlled laboratory tests, it is unviable to compute individual prior beliefs and dynamic state probabilities for most naturalistic tasks. However, adaptive solutions like PbAC do not require complex computational estimations or controls to be implemented; rather, our findings suggest that diverse predictive processes can be captured and modulated using real-time data analytics and task customisation. Hence, this approach could provide detailed insight about a user’s neuropsychological state; perhaps as a method of monitoring performance (e.g. surprisal and re-framing responses in pilots: Rankin et al. 2016), augmenting therapies (e.g. for autistic people: Haker et al. 2016), or improving empirical protocols (e.g. to better understand perceptual atypicalities in XR: Harris et al. 2019).

On the other hand, PbAC could also be developed as a method of optimising XR experiences: for instance, to try and boost engagement (e.g. by transforming the salience of sensory cues; Bontchev and Vassileva 2016), decrease cybersickness (e.g. by reducing discrepancies between expected and perceived sensory information; Chang et al. 2020), or improve skill performance (e.g. by augmenting prior beliefs about an opponent in sport; Wang et al. 2019). These applications could be informed by applied theories of perception and action which outline possible mechanistic pathways for achieving a desired state or sensory experience. For instance, the movements and position of an avatar’s limbs could be adapted based on real-time estimates and modelling of prediction error, in an attempt to promote embodiment and immersion (in line with models of presence and place illusion: e.g. Witmer & Singer, 2005; Slater, 2009) or even to direct attention towards favourable stimuli during training (e.g. external as opposed to internal cues in motor learning; see Wulf 2013). In this regard, next-generation adaptive XR systems could be designed through the lens of PbAC and its personalised modelling capabilities for translation across various fields and domains. Training and rehabilitation sectors may represent the most fruitful avenue for such design endeavours, given the capacity for developers to exploit in-situ computational modelling and biomarkers (e.g. from gaze/pupillometry data or HGF parameters like ε₂ or α₂) to adapt simulated task conditions (e.g. to align with a user’s sensory profile, adjust their learning dynamics, or promote a given motor response).

Clearly, these potential applications remain speculative at this stage and caution must be taken when generalising our initial ‘proof of concept’ results. Indeed, challenges may arise when developing PbAC functions for complex XR environments, where numerous interacting sensorimotor processes and cue-outcome associations are involved (e.g. in multi-player games or applications that contain simultaneous targets, goals, and social inputs). Our study utilised a relatively simple XR environment that was specifically built for assessing a user’s dynamic predictive beliefs and error responses via eye tracking analyses. PbAC may require significant re-packaging and designing for applications where eye tracking data is more ambiguous and/or noisy to analyse, such as tasks involving rapid target occlusion, cluttered visual workspaces, and competing attentional demands. Nonetheless, our study demonstrates a replicable design approach for tasks that contain clear, modifiable cue-outcome associations and a capacity to measure dynamic state beliefs or user expectations. In these contexts, our data show that goal-directed motor actions, sensory sampling processes, and physiological surprise responses can be modulated by PbAC, while established patterns of individual behaviour are replicated (e.g. correlations between autistic-like traits and predictive processing outcomes: see Supplementary File 2).

Still, there are limitations associated with our ‘proof of concept’ methodology that must be considered. Most notably, our study’s virtual racquetball task consists of relatively simple and artificial conditions, especially in comparison to the more applied scenarios discussed above. This not only allowed key task variables to be controlled (e.g. headset tracking quality, sensory stimuli, and movement demands), but it also made real-time data computations relatively straightforward (e.g. eye tracking analyses could simply focus on the side of the court that participants were gazing towards, with minimal ‘real-time’ preprocessing or cleaning of data required). In its current form, PbAC assumes a tight coupling between a user’s overt gaze and predicted outcomes; however, multimodal inferences may be required in future iterations (e.g. incorporating head pose, electromyography and/or kinematic data signals), for contexts where suboptimal gaze-belief coupling emerges. Thus, it is necessary to determine the generalisability of PbAC within more complex and naturalistic environments, where inferences about user predictions and surprisal may be more challenging to index. Recent eye-tracking analyses have offered promise in this regard, with insight about individual prior beliefs, uncertainty estimates, and surprisal being retrieved across diverse settings (e.g. in sport: Beck et al., 2024; driving simulation: Engström et al. 2024; and clinical research: Arthur et al. 2021).

A further limitation of the study relates to the relatively low number of trials used and the lack of counterbalancing for the ordering of study blocks. These factors may have particularly impacted upon model-based estimations, as some reliably observed effects (e.g. higher ε_2_ and ε_3_ values on trials with probabilistically unlikely outcomes) did not emerge in our data. While research has shown that prior beliefs can be learned in as little as 10–20 trials (Kwon and Knill 2013; Pasturel et al. 2020; Verstynen and Sabes 2011), it is apparent that most protocols in the literature have utilised a larger number of repetitions. Evidently, it is not always feasible to employ large amounts of trials for naturalistic movement-based tasks; however, future research may wish to explore the potential for repeated within-trial measurements (e.g. by calculating dynamic prior beliefs and prediction error values across extended timeseries data, as in Perrykkad et al. 2021). Moreover, since the ordering of conditions could not be counterbalanced in this study, future research could examine whether changes in motivation and fatigue potentially confound sensorimotor behaviours and model outcomes. No general reductions in task performance or tracking accuracy were discernible between blocks in our study, but previous research has shown that gaze and pupil responsivity can be affected by these variables in naturalistic movement-based tasks (e.g. Wykowska et al. 2013; Loiseau-Taupin et al. 2021; Deng et al. 2025). Relatedly, although no participants reported experiencing any adverse effects when using VR, it is possible that minor levels of discomfort or cybersickness will have influenced our study conditions and results (e.g. by impacting on pupil dilation responses and task motivation). As such, we recommend that researchers measure cybersickness formally in future studies, using standardised tools (e.g. the Simulator Sickness Questionnaire; Kennedy et al. 1993).

In conclusion, the present study provides initial proof of concept for PbAC and its potential application as a method of monitoring and modulating predictive processing operations. Our findings demonstrate that this novel technological solution is able to capture diverse sensorimotor responses and their underlying neuropsychological mechanisms, in accordance with Bayesian and active inference models of the brain. Crucially, such computations can be performed within adaptive XR simulations, in a manner that allows for dynamic, personalised modifications to sensory stimuli and environmental conditions. Results indicate that these modifications can be aligned with the fundamental predictive processes that are proposed to underpin human perception, action and learning functions. Future research must now be conducted to develop the novel PbAC concept into naturalistic, technologically matured XR solutions, which can be exploited by wide-ranging academic and applied industries.

## Supplementary Information

Below is the link to the electronic supplementary material.

Supplementary Material 1

## Data Availability

All relevant data and code are available online from https://osf.io/37xjw/. A Unity package relating to the study’s virtual racquetball task is available on this page and contains all relevant PbAC code and scripts. However, please note that this code has been specifically built for our virtual racquetball task and would require modification for use in other Unity projects and XR environments.
