# Supplementary material for: Prediction-based attention computing: a proof of concept study
Source: Virtual Real. 2026 Jan 19;30(1):44. doi: 10.1007/s10055-025-01307-w (PMC12855329; doi:10.1007/s10055-025-01307-w)
Supplement: Supplementary file 1 — Supplementary Material 1 [file 10055_2025_1307_MOESM1_ESM.pdf]

Supplementary File 1: Participant Flow Diagram

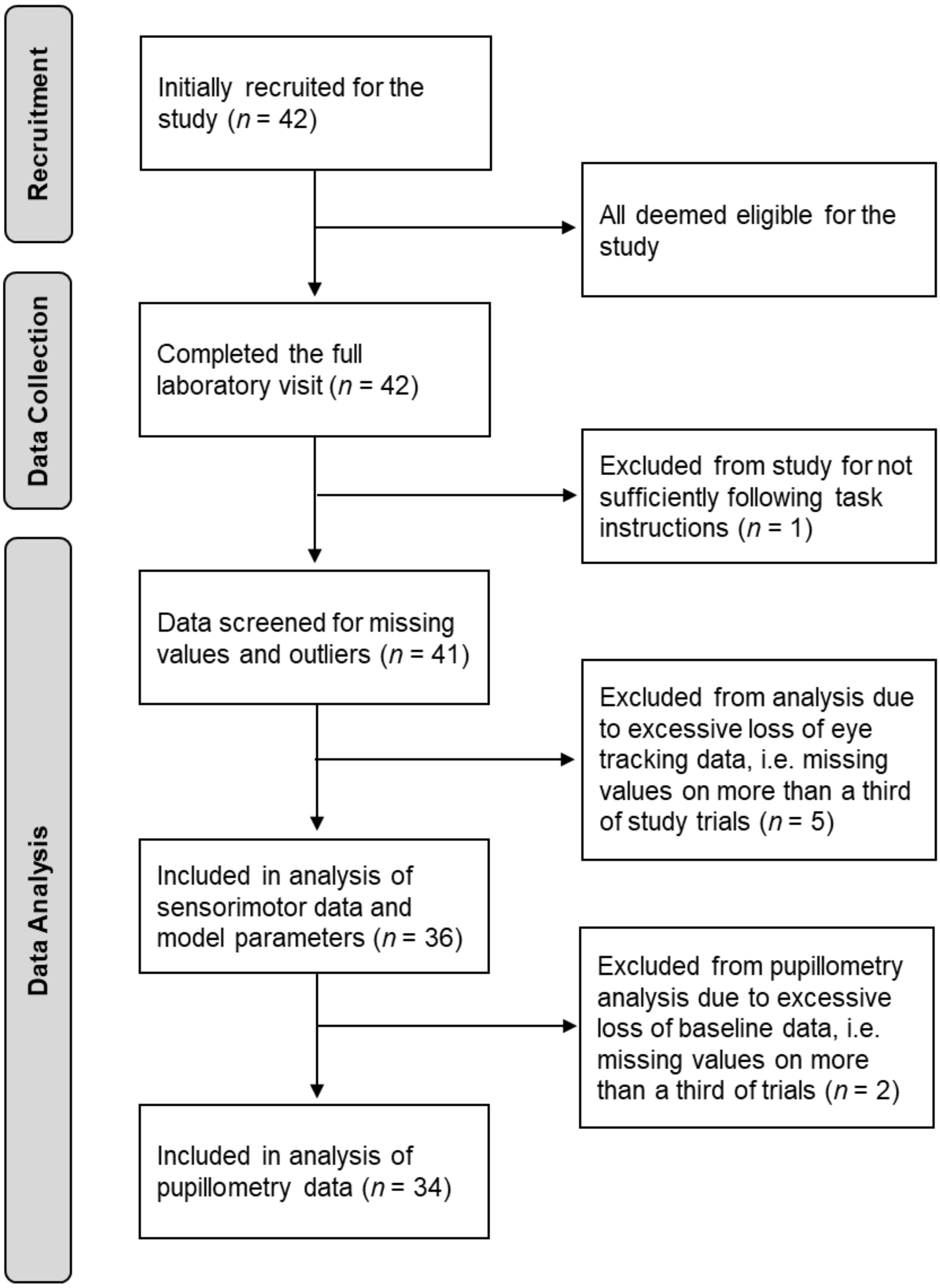

Supplementary Figure 1. Diagram outlining the number of participants included in the study, following initial recruitment, data collection, and data analysis phases of the research.

Supplementary File 2: Exploratory Analyses

The present study found that predictive sensorimotor responses were sensitive to adaptive PbAC conditions. However, substantial variability was evident between participants, as shown in Figures 4-5. Given that PbAC is specifically designed to capture personalised behaviours, these exploratory analyses tested whether the novel computing method can accurately represent the natural differences in predictive processing that exist between individuals.

Specifically, we examined the degree to which PbAC conditions captured underlying differences in predictive processing that exist between individuals with varying autistic-like traits. Autistic-like traits refer to a spectrum of characteristics that resemble common strengths and difficulties associated with autism, which are said to be distinct from a clinical diagnosis and distributed across the general population (see Ruzich et al., 2015). Previous studies have shown that people with higher autistic-like traits can exhibit a reduced distinction between expected and unexpected cues, both in their neural (Ewbank et al., 2014) and behavioural (Arthur et al., 2021; Lawson et al., 2017) responses. Accordingly, participants in our study were presented with the 50-item AQ (see *Methods*), a well-established tool that has been widely studied in both clinical and general populations (Baron-Cohen et al., 2001; Ruzich et al., 2015). The AQ examines five key domains associated with autism, namely: social skills, attention switching, attention to detail, communication, and imagination. Participants self-reported the degree to which they “Definitely agree”, “Agree”, “Disagree”, or “Definitely disagree” with 50 itemised statements, and an overall score was then computed for each individual (possible range: 0–50). Based on the previous research evidence, described above, we hypothesised that higher AQ scores would correlate with reduced differences (Δ) in predictive sensorimotor behaviours and pupil-based surprise during PbAC conditions.

There was a wide variation in autistic-like traits recorded for our study sample, with AQ scores ranging from 8 to 38 (*M*: 19.85 ± 6.62). Notably, exploratory analyses highlighted weak negative correlations between these varying AQ scores and the magnitude of difference between expected and unexpected trials for predictive sensorimotor outcomes (see Supplementary Figure 2). Specifically, participants with higher autistic-like traits showed relatively lower Δ interception rate (R = -.38, p = .02, BF_{10} = 2.42) and Δ gaze tracking error (R_s = -.40, p = .02, BF_{10} = 2.21; as in Arthur et al., 2021), although null relationships emerged between AQ scores and peak pupil diameter (for unexpected trials only: R_s = -.20, p = .25, BF_{10} = 0.53). These results indicate that the PbAC conditions were able to capture individual differences in predictive processing that correspond with varying autistic-like traits across the study sample.

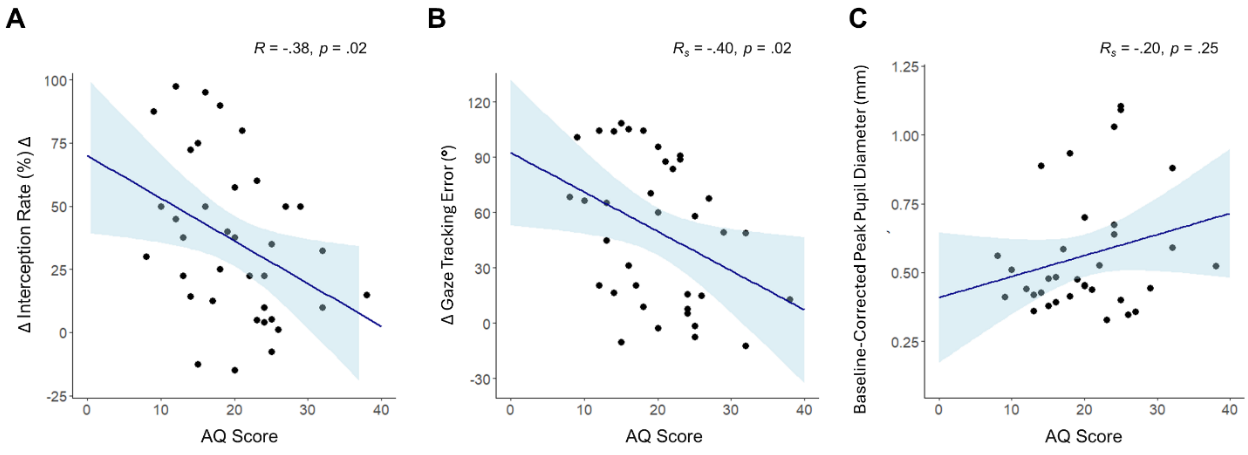

Supplementary Figure 2. Relationships between predictive sensorimotor outcomes and autistic-like traits, as measured on the 50-item Autistic Quotient (AQ).

Supplementary File 3: Starting Parameters for the Computational Models

In each of the Hierarchical Gaussian Filter (HGF) and associative learning models described in Section 2.3.4, a perceptual component (i.e. a person's beliefs of their environment) and a response component (i.e. linking these beliefs to their actions) were combined to estimate participants' learning and decision processes. Here, a series of free parameters were optimised via maximum-a-posteriori estimation, to fit the experimental data that we wished to model. As such, a series of starting parameters were entered into each model, which would subsequently vary to accommodate our observed study data. This approach follows the "observing the observer" framework outlined in Daunizeau et al (2010), and our starting parameters were determined using the same statistical procedures outlined in Harris et al (2023).

More specifically, prior means in the HGF models (Supplementary Table 2) were determined by running simulations through real trial sequences in the experiment, while corresponding free value beliefs in the associative learning models (α, h, and v in Supplementary Table 1) were set at neutral starting values. In line with Harris et al.'s (2023) previous study, these starting beliefs were given a wide variance in all five models (e.g., see values of 8 for ω, ϑ, and μ in Supplementary Table 2), and Kappa (κ) was fixed at 1 with minimal variance in the HGF variants. These starting parameters were chosen to reduce model complexity while allowing for individual differences in the data, as described in Section 2.3.4 (and more extensively in Harris et al., 2023).

Supplementary Table 1. Prior means (and variances) for the Associative Learning models

R-W model	α = 0.5 (1)	v = 0.5 (1)
Sutton K1 model	h = 0.5 (1)	v = 0.005 (16)

Supplementary Table 2. Prior means (and variances) for the Hierarchical Gaussian Filter models

Model	κ**	ω	ϑ	$\vartheta 2$	$\mu 2$	$\sigma 2$	$\mu 3$	$\sigma 3$	$\mu 4$	$\sigma 4$
HGF2	1 (0)	-6.95 (8)	-4 (0)	-	1.33 (8)	0.07 (1)	-0.05 (0)	1 (1)	-	-
HGF3	1 (0)	-6.95 (8)	-4 (8)	-	1.33 (8)	0.07 (1)	-0.05 (8)	1 (1)	-	-
HGF4	1 (0)	-6.95 (8)	-4 (8)	-2 (8)	1.33 (8)	0.07 (1)	-0.05 (8)	1 (1)	1 (8)	1 (1)

Supplementary File 4: Additional Figures and Data Illustrations

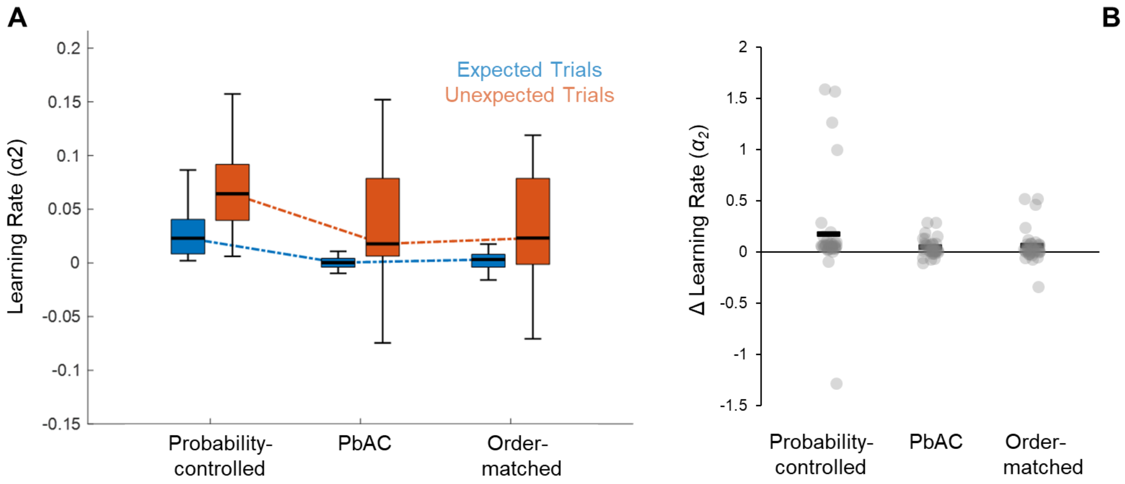

Supplementary Figure 3. Model-derived learning rates during each of the three study conditions. Bars in panel ***A*** and ***C*** represent the median averages (black lines) and the upper and lower quartiles for each condition. Whiskers represent the minimum and maximum data values, excluding outliers. In panels ***B*** and ***D***, circles represent mean values for each individual participant, while black lines represent the mean averages for each overall condition. PbAC: Prediction-based Attention Computing.
